# The Genetic Factors Influencing Cardiomyopathies and Heart Failure across the Allele Frequency Spectrum

**DOI:** 10.1007/s12265-024-10520-y

**Published:** 2024-05-21

**Authors:** Srinjay Mukhopadhyay, Prithvi Dixit, Najiyah Khanom, Gianluca Sanghera, Kathryn A. McGurk

**Affiliations:** 1 National Heart and Lung Institute, Imperial College London, LMS Building, Hammersmith Campus, London, UK; 2 MRC Laboratory of Medical Sciences, Imperial College London, London, UK; 3 School of Medicine, Cardiff University, Wales, UK

**Keywords:** Heart failure, Genetics, Cardiomyopathy

## Abstract

Heart failure (HF) remains a major cause of mortality and morbidity worldwide. Understanding the genetic basis of HF allows for the development of disease-modifying therapies, more appropriate risk stratification, and personalised management of patients. The advent of next-generation sequencing has enabled genome-wide association studies; moving beyond rare variants identified in a Mendelian fashion and detecting common DNA variants associated with disease. We summarise the latest GWAS and rare variant data on mixed and refined HF aetiologies, and cardiomyopathies. We describe the recent understanding of the functional impact of titin variants and highlight *FHOD3* as a novel cardiomyopathy-associated gene. We describe future directions of research in this field and how genetic data can be leveraged to improve the care of patients with HF.

**Graphical Abstract:**

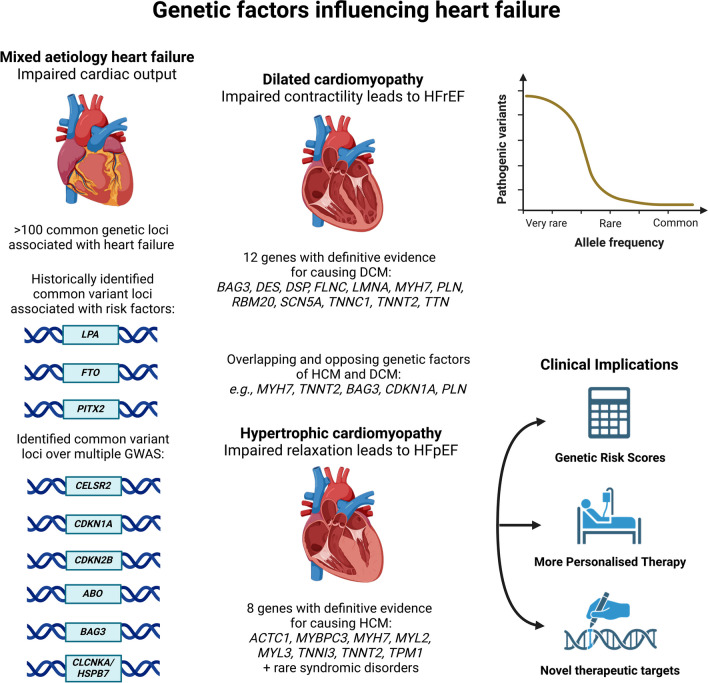

## Heart Failure, Cardiomyopathies, and Genetic Analyses

Heart failure (HF) is a heterogeneous cardiac condition arising from functional or structural abnormalities of the heart, resulting in impaired ventricular filling and cardiac output [1]. It is highly prevalent, affecting over 64 million individuals worldwide [1]. Despite therapeutic advances, the risk of HF-related morbidity and mortality remains high, with an average 5-year survival rate of approximately 50% [2]. The estimated heritability (h^2^) of HF, the amount of variation in HF due to genetic factors, is substantial (h^2^ ~ 18%-26% [3]), and elicited studies to identify disease-associated genomic loci to inform novel therapeutic strategies [4–6].

Cardiomyopathies are structural or functional myocardial conditions that are an established cause of HF. They present in the absence of secondary causes of HF such as hypertension, valvular disease, coronary artery disease, or congenital heart disease. Patients with cardiomyopathy may present with symptoms of HF with reduced ejection fraction (HFrEF) or preserved ejection fraction (HFpEF).

**Fig. 1:**
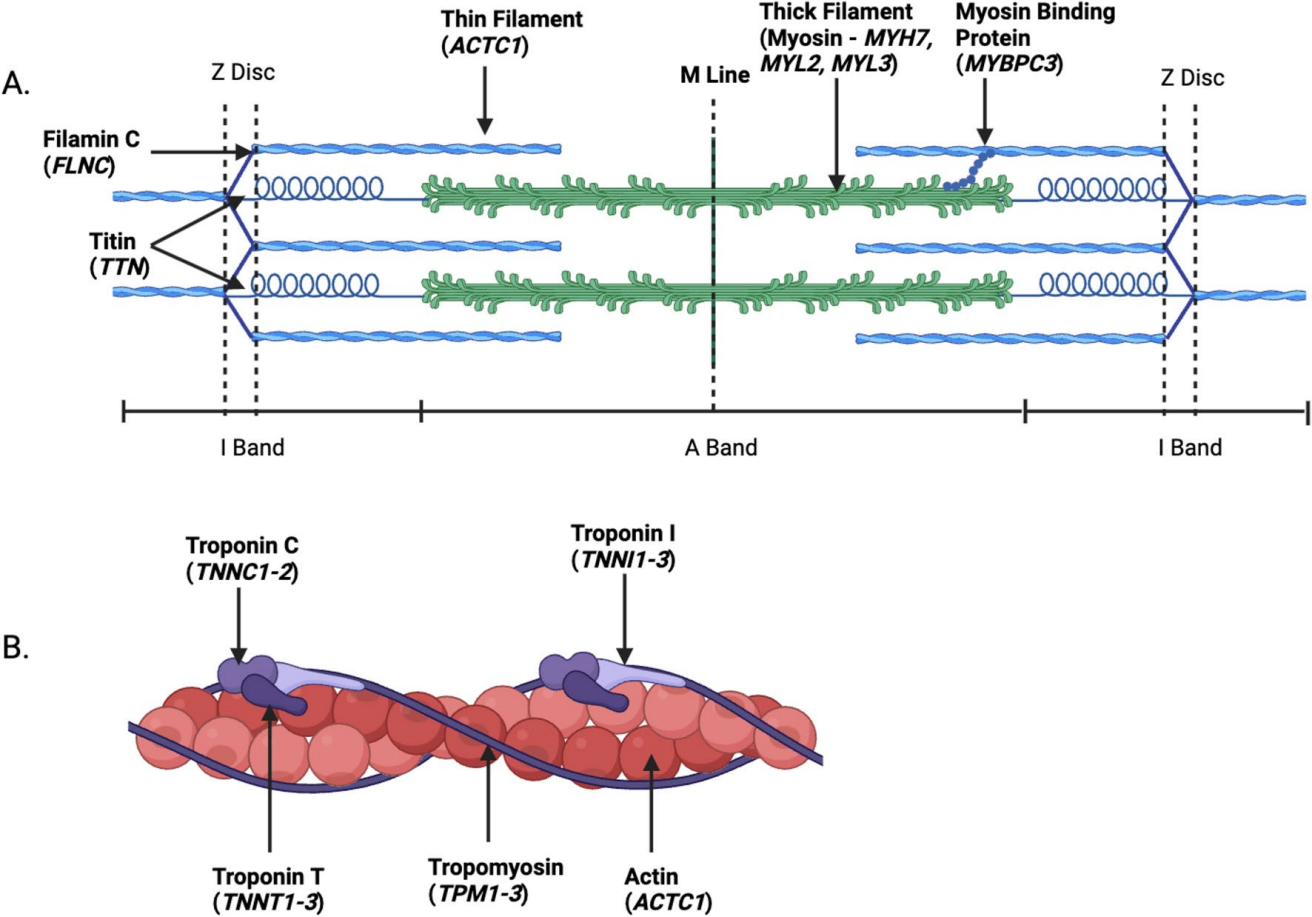
Components of the sarcomere, the basic contractile unit of a cardiomyocyte. **A** The labelled genes of the cardiac sarcomere and **B** a depiction of the actin-tropomyosin complex. The image was created with BioRender.com

Many of the proteins transcribed from genes that are curated to have definitive evidence of causing cardiomyopathies are found in the sarcomere of cardiomyocytes. The sarcomere is the basic contractile unit of the cardiomyocyte [7]. It is composed of thin actin filaments and thick myosin filaments (Fig. 1) [8, 9]. The sliding filament theory describes cardiac contraction occurring through the motion of actin filaments sliding past myosin filaments and their interaction [10]. The tropomyosin and troponin complex are fundamental components of the sarcomere, which function as regulatory proteins and control the interaction of actin and myosin, respectively. [11] Variants in sarcomeric genes, such as myosin binding protein (*MYBPC3*)*,* myosin heavy chain (*MYH7*)*,* and titin (*TTN*)*,* are major genetic determinants of cardiomyopathies [12], with ~ 30% of patients heterozygous for a sarcomere mutation [13, 14].

Dilated cardiomyopathy (DCM) is characterised by the dilation of the left or both ventricles of the heart, which reduces myocardial contractility leading to systolic dysfunction [15]. It is regarded as one of the primary contributors to HFrEF and is the leading indication for heart transplantation globally [16, 17]. Hypertrophic cardiomyopathy (HCM) is characterised by left ventricular wall thickness [18]. The concentric hypertrophy accompanied by fibrosis and myocardial disarray leads to impaired myocardial relaxation and diastolic dysfunction. HF is prevalent in most patients with obstructive HCM (those with left ventricular outflow tract obstruction due to septal hypertrophy) and ~ 10% of patients with non-obstructive HCM [16]. HFpEF is the most common HF phenotype amongst HCM patients as a result of decreased left ventricular compliance secondary to increased wall thickness. [19]

Genetic research on cardiomyopathies has aided the determination of the mechanisms underlying abnormal cardiac function leading to HF. Historically, linkage analyses of familial cardiomyopathies identified causal DNA variants inherited in a Mendelian fashion [20]. Linkage analysis refers to statistical methods used to map a gene to the region of the chromosome in which the gene is located [21]. Improved genome-wide sequencing technologies have allowed for unbiased assessments of the genome; and genome-wide association studies (GWASs) of common DNA variants [22, 23]. Genotyping microarray data is used as input for GWAS and measures common variants sporadically across the genome. GWASs map the polygenic architecture of HF by comparing the alleles of single nucleotide polymorphisms (SNPs) present in patients with HF to those of non-HF or reference populations [24]. Identification of the common variants with alleles that are significantly increased or decreased in patients with HF has implicated polygenicity and identified modifying genetic factors, in disease [25]. The heterogeneous presentation of HF suggests a diverse genomic landscape and we discuss some examples in this review [26–28].

The recent European Society of Cardiology guidelines (2023) for the management of cardiomyopathies recommend genetic testing in individuals with cardiomyopathies to inform prognosis, treatment selection, reproductive advice, and psychological benefit [29]. There are different methods available to sequence the genome depending on genome coverage and cost. The use of next-generation sequencing (NGS) has revolutionised genomic research, allowing for the human genome to be sequenced within 20 minutes cost-effectively [30, 31]. Whole-genome sequencing (WGS) determines the entire human genome, encompassing the non-protein coding (denoted “non-coding”, but codes for some RNA transcription and other regulators) and protein-coding regions. In contrast, whole-exome sequencing (WES) determines the exome, encompassing only the protein-coding exons [32]. Although WES only determines the coding regions, the rationale for its utility is that genetic variants within the coding region account for the most significant effect variants related to diseases [33]. WGS and WES improve the yield of a cardiomyopathy genetic diagnosis when compared to genetic testing using targeted panels (i.e., selected gene panels that only sequence disease-associated genes). While panel sequencing is more rapid, WGS and WES allow for revisiting the genetic data upon identification of novel cardiomyopathy-associated genes from a once-off effort (e.g., the recent identification of disease-causing filamin C [*FLNC*]) but at an increased expense, computational time, bioinformatics, and computational requirement [34].

WES and WGS allow for the discovery of novel disease-causing variants [33] but in a clinical setting, the identification of genetic variants with insufficient evidence of pathogenicity (variants of uncertain significance [VUSs]) can confuse patients and are less useful to the clinician and healthcare programs. The American College of Medical Genetics and Genomics created guidelines for the interpretation of sequence variants based on variant evidence (e.g., population data, computational data, functional data, and segregation data; results reported as (likely) benign, VUS, or (likely) pathogenic). The ClinGen Consortium Gene-Disease Clinical Validity curation process allows experts to evaluate the strength of evidence supporting or refuting a claim that variation in a particular gene causes a particular monogenic disease (results reported as definitive, strong, moderate, limited or weak evidence for causing disease) [35, 36].

This review explores the common and rare variants associated with mixed aetiology HF. We highlight the common variants associated with HCM and DCM from recent large GWAS before exploring the implications of rare variants and the function of pathogenic variants that have a causal relationship with cardiomyopathies (Graphical Abstract). We describe truncating variants in the titin gene, the most common genetic cause of DCM, and recent mechanistic insights into how they influence cardiac function in DCM, and describe a recent HCM-associated gene, *FHOD3*, before highlighting future directions in the field and how genetic data may be leveraged to improve the care of patients with HF.

## Common DNA Variants Associated with Heart Failure

Cappola et al. (2010) conducted an early HF GWAS-like study, profiling 30,000 SNPs in 2,000 genes thought to be associated with cardiovascular disease [37]. They compared the genotypes of patients with advanced HF to controls without HF, and SNPs that reached statistical significance were tested in a replication cohort (statistical significance is usually a Bonferroni Correction for the number of tests (SNPs), e.g., standard GWAS threshold is *P* = 5 × 10^–8^; a P-value of 0.05/1 million independent SNPs). Two significant SNPs were identified; one near *HSPB7* encoding a heat shock protein and one near *FRMD4B* (FERM domain containing protein B).

It was postulated that carrying these variants individually only translated to a small, clinically insignificant, increased risk of HF. Variants observed enough times for statistical power to identify a difference in the population have alleles that are common and usually found in > 1% of the population. HF prevalence is ~ 1% and so such common variants individually cannot have a large effect in causing HF [38]. Genome-wide rare variant association studies (RVAS) of rare genetic variants (i.e., found in one patient) with predicted large effects on disease are usually grouped by a certain category to allow for statistical testing (e.g., by gene and variant consequence to the protein for a specific gene; any rare *MYBPC3* missense variants).

Shah et al. (2020) conducted a meta-analysis of HF GWAS using data from 29 studies encompassing 47,309 cases and 930,014 controls [39]. 11 genomic loci reached genome-wide significance (*P* < 5 × 10^−8^; Table 1 [39–42]). Many of the loci identified have strong associations with cardiovascular disease risk factors such as *FTO* (fat mass and obesity-associated; associated with BMI), *PITX2* (the PITX2 transcription factor; associated with atrial fibrillation [AF]), and *LPA* (lipoprotein(a); associated with coronary artery disease [CAD]). To determine whether these variants were associated with HF independent of other risk factors, the authors undertook a multi-trait conditional and joint analysis. Conditioning for AF, the authors found that there was a > 50% attenuation in HF risk for *PITX2* and other AF-associated loci (*SYNPOL2, KLHL3,* and *AGAP5*) remained independently associated with HF. Similarly, conditioning for CAD fully attenuated the HF risk for *LPA*.

Levin et al. (2022) conducted a large multi-ancestry GWAS including 115,150 HF cases and 1,550,331 controls, leveraging data from various biobanks (HERMES, Penn Medicine Biobank, eMERGE, Mount Sinai BioMe, Geisinger DiscovEHR, FinnGen, and the Global Biobank Meta-Analysis Initiative) [40]. They identified 47 disease-associated loci (Table 1) of which 39 were validated in an independent replication cohort. The authors also evaluated the genetic correlations (the genetic similarity of two traits) between cardiac imaging phenotypes (left ventricular end-systolic volume, end-diastolic volume, and ejection fraction) and HF. They found a substantial genetic correlation between HF and left ventricular end-systolic volume (correlation coefficient R = 0.36; *P* = 3.73 × 10^−16^).

**Table 1:** The loci of common variants implicated in HF through genome-wide association studies

GWASs reporting genes associated with heart failure							
Nearest Gene to the Lead SNP identified	Shah et al. [39]	Levin et al. [40]	Joseph et al. [41]	Henry et al. [42] [Unclassified HF]	Henry et al. [42] [Non-Ischaemic HF]	Henry et al. [42] [Non-Ischaemic HFrEF]	Henry et al. [42] [Non-Ischaemic HFpEF]
*CELSR2*	*✔*		*✔*				
*PITX2*	*✔*	*✔*	*✔*		*✔*		
*KLHL*	*✔*						
*CDKN1A*	*✔*	*✔*	*✔*			*✔*	
*LPA*	*✔*	*✔*	*✔*	*✔*			
*CDKN2B*	*✔*	*✔*	*✔*	*✔*			
*ABO*	*✔*		*✔*		*✔*		
*SYNPO2L*	*✔*	*✔*					
*BAG3*	*✔*		*✔*			*✔*	
*ATXN2*	*✔*						
*FTO*	*✔*	*✔*	*✔*		*✔*		
*RP11-116D17.1*		*✔*					
*NPC1*		*✔*					
*ZFHX3*		*✔*					
*HDGFL1*		*✔*					
*SURF6*		*✔*					
*CLCNKA/HSPB7*		*✔*	*✔*			*✔*	
*IRAK1BP1*		*✔*					
*FAM133B*		*✔*					
*STRN*		*✔*				*✔*	
*BAZ1A*		*✔*					
*SRR*		*✔*					
*GNPDA2*		*✔*			*✔*		
*KLHL3*		*✔*					
*PMAIP1*		*✔*		*✔*			
*TMEM18*		*✔*					*✔*
*GTF2I*		*✔*	*✔*				
*HSD17B12*		*✔*					
*HECTD4*		*✔*					
*SH2B3*		*✔*					
*DMRTA2*		*✔*					
*USP36*		*✔*					
*OR2A2*		*✔*					
*POM121C*		*✔*					
*CACNB2*		*✔*					
*ASXL3*		*✔*					
*CHMP3*		*✔*					
*TTC39A*		*✔*					
*NFIA*		*✔*				*✔*	
*KCNIP4*		*✔*					
*TUBA3C*		*✔*					
*ORC5*		*✔*					
*ZNF280A*		*✔*					
*COX7C*		*✔*					
*E2F6*			*✔*				
*ABHD5*			*✔*				
*CAMK2G*			*✔*				
*NFAT5*			*✔*				
*SMG6*			*✔*				
*PNMT/PGAP3*			*✔*				
*YPEL2*			*✔*				
*BPTF*			*✔*				
*18q12.2*			*✔*				
*MAP3K7CL*			*✔*				
*LDLR/SMARCA4*				*✔*			
*WNT3*				*✔*			
*HIC1*				*✔*			
*CMIP*				*✔*			
*HAGHL/NARFL*				*✔*			
*ACTN1*				*✔*			
*COL4A1*				*✔*			
*ALDH2*				*✔*			
*LPL*				*✔*			
*ZNF318*				*✔*			
*NKX2*				*✔*			
*PFDN1*				*✔*			
*NBEAL1*				*✔*			
*ABCG5*				*✔*			
*PSRC1*				*✔*			
*PPAP2B*				*✔*			
*CASZ1*				*✔*			
*XRN2*					*✔*		
*ERBB2*					*✔*		
*CORO1A*					*✔*		
*ABHD17C*					*✔*		
*RFX4*					*✔*		
*C9orf3*					*✔*		
*TNKS*					*✔*		
*CDK6*					*✔*		
*GTF2IRD1*					*✔*		
*RSPO3*					*✔*		
*SNRPC*					*✔*		
*RBM27*					*✔*		
*NR3C1*					*✔*		
*CXXC5*					*✔*		
*NAF1*					*✔*		
*EXOSC9*					*✔*		
*DLIM5/SMARCAD1*					*✔*		
*SLC4A7*					*✔*		
*ATP1B1*					*✔*		
*FUBP1*					*✔*		
*CDKN2C*					*✔*		
*FOXJ3*					*✔*		
*BACH1*						*✔*	
*HLF*						*✔*	
*KLF12*						*✔*	
*FUT11*						*✔*	
*CAMK2D*						*✔*	
*SLC6A6*						*✔*	
*CAND2*						*✔*	
*SGOL2*						*✔*	
*ACTN2*						*✔*	
*FLNC*						*✔*	
*ADAMTS7*						*✔*	
*CCDC178*						*✔*	
*GALNT8*							*✔*
*IGFBP7*							*✔*

The table references genome-wide association studies (GWASs) and the nearest gene of lead SNPs associated with unclassified HF. For studies which identified multiple loci, only those with the strongest association with unclassified HF are shown. For the most recent GWAS by Henry et al. (2023), we also report the genes associated with non-ischaemic heart failure, non-ischaemic heart failure with reduced ejection fraction, and non-ischaemic heart failure with preserved ejection fraction

Both GWAS meta-analyses identified the *PITX2* locus to have the strongest association with HF. The PITX2 transcription factor plays an important role during embryonic development of the heart [43]. Loss of function variants (e.g., stop gained, frameshift, and essential splice variants) in *PITX2* have been associated with atrial structural and functional remodelling leading to an increased risk of arrhythmogenesis [44].

Joseph et al. (2022) conducted a GWAS using the US Million Veteran Program (MVP) to assess the separate genetic architecture of HFrEF and HFpEF [41]. They identified 13 genetic loci associated with HFrEF, compared to only one locus for HFpEF (near the *FTO* gene). The estimated heritability of the HF subgroups was lower for HFpEF than HFrEF (h^2^ = 1.9% and 3.1%, respectively). There is a need for sub-phenotyping of HFpEF patients as the current classification encompasses the final manifestation of multiple diverse aetiologies. More refined phenotypic definitions of HFpEF may improve the statistical power for genetic discovery in future studies or identify modifying environmental and non-genetic factors.

Henry et al. (2023) reported a GWAS meta-analysis on a cohort of 1.9 million ancestrally diverse people, including 153,174 cases of HF [42]. They identified 66 genetic susceptibility loci across HF subtypes (Table 1). The *IGFBP7* gene was identified to have a significant association with non-ischaemic HFpEF. *IGFBP7* encodes an anti-angiogenic factor and has been implicated in cardiomyocyte senescence and cardiac remodelling [45]. Its association with HFpEF raises the possibility of altered tissue homeostasis and renewal contributing to the development of this phenotype. The estimated heritability of HF explained by common variants in this study was 5.4% for unclassified HF. Again, the heritability of non-ischaemic HFrEF was substantially greater than that of non-ischaemic HFpEF (h^2^ = 11.8% and 1.8%, respectively). The authors assessed the genetic correlation between HF subtypes; HFrEF and HFpEF had a substantial genetic correlation (r_g_ = 0.42; SE = 0.18).

Many of the genetic loci identified in mixed aetiology HF GWAS to date are those associated with HF risk factors such as AF and CAD. Importantly, the myriad of conditions which lead to HF (and some which may be genetically opposing, e.g., HCM and DCM) limit the statistical power to detect common variants associated with the disease. There is a need for better sub-grouping of patients with HF, perhaps based on aetiology (e.g. ischaemia or valvular disease leading to HF), to better investigate genetic associations, alongside the analysis of larger biobanks.

## Common DNA Variants Associated with Hypertrophic Cardiomyopathy

It is well-accepted that the initial monogenic sarcomere mutation hypothesis alone for HCM is outdated [46–48]. This is especially true considering that ~ 60% of HCM cases detected at the onset of disease are genotype negative and many without family history [49]. Sarcomere-negative HCM is a complex, likely polygenic trait. A GWAS by Harper et al. (2021) confirmed a markedly greater common variant genetic contribution to sarcomere-negative HCM than sarcomere-positive/Mendelian HCM (*h*^2^ = 34% and 16%, respectively) [27]. This supports the hypothesis that common variants in combination may have a causal role in HCM.

A genetic risk score (GRS) was leveraged to predict the odds of HCM from 27 HCM-associated SNPs. To increase statistical power, the GRS was estimated from a meta-analysis of three independent HCM populations of European descent with 1,769 cases and 39,828 controls. A protective effect against HCM was observed for individuals in the lowest quintile. HCM risk more than doubled for individuals in the highest quintile (i.e., participants with risk alleles) compared to the central 60% of the cohort analysed [27].

Variants in the cyclic-dependent kinase inhibitor 1A (*CDKN1A*) locus have been significantly associated with HCM (rs3176326) [27, 50]. The protein is involved in cell cycle arrest. A recent GWAS meta-analysis concluded that this kinase holds a direct phenotypic effect in the heart, causing reduced left ventricular systolic function and cardiomyocyte cell cycle arrest [39]. An in-vivo model of *CDKN1A* knockout mice developed cardiac hypertrophy and HF [51]. A phenome-wide association study (PheWAS; comparing a trait in individuals with or without a reported clinical outcome) further identified the *CDKN1A* locus as significantly associated with HCM and ECG traits (PR interval and QRS duration) [52].

Tadros et al. (2023) conducted a GWAS of over 5,000 HCM cases and over 30,000 UK Biobank participants with cardiac magnetic resonance imaging available for analyses [53]. 33 loci and 35 novel loci were associated with HCM. When stratified by sarcomere-positive and sarcomere-negative HCM, an additional 1 locus and 4 loci were identified, respectively. A locus on chromosome 11 (near *MYBPC3*, a definitive-evidence HCM gene) associated with HCM and sarcomeric HCM, but not sarcomere-negative HCM, suggesting the locus is in linkage disequilibrium with founder *MYBPC3* variants. Higher heritability was estimated for genotype-negative HCM compared to genotype-positive HCM (h^2^ = 29% and 16%, respectively), reflecting the complex genetic influence underlying HCM [27].

## Common DNA Variants Associated with Dilated Cardiomyopathy

Pirruccello et al. (2020) undertook a GWAS of cardiac imaging traits in the UK Biobank cohort to identify genetic loci influencing DCM risk [54]. Utilising the cardiac magnetic resonance imaging (MRI)-derived left ventricular measurements of end-diastolic and systolic function from 36,000 participants, 45 previously unreported genome-wide significant loci were identified. Common variants at 18 loci were identified near Mendelian cardiomyopathy genes; 12 were newly discovered. GRS based on the cardiac MRI parameters demonstrated that common genetic variation influenced cardiac function and structure. Notably, the GRS for indexed left ventricular end-systolic volume (LVESVi), comprising 28 SNPs, robustly predicted DCM. However, the findings were limited to individuals of European ancestry.

Garnier et al. (2021) conducted a GWAS evaluating over 2,700 sporadic DCM cases [55]. The findings identified robust replication of two out of three novel genome-wide significant loci found to increase the risk of sporadic DCM by a third. This includes *SLC6A6* which encodes a taurine transporter, a nonessential amino acid which maintains cellular integrity and is found in very high concentrations in the heart and muscle [56–59]. A recent article in *Science* has identified anti-aging effects of this amino acid but a clinical trial is needed to investigate whether supplementation improves the symptoms of patients with DCM [60].

Zheng et al. (2023) conducted the largest DCM GWAS to date comprising over 14,255 DCM cases from the HERMES consortium [61]. Through gene prioritisation strategies such as pathway enrichment analysis and utilisation of genetically correlated CMR traits from UK biobank, 80 loci associated with DCM were discovered. Genes involved in cell-to-cell adhesion and cell-to-matrix interaction were found to play a role in DCM pathogenesis, alongside established biological pathways such as contractile and cytoskeletal functions. An example novel gene is *SSPN*, encoding sarcospan, a core component of the tetrameric dystrophin-glycoprotein complex (DGC) typically associated with muscular dystrophies. A preclinical study by Parvatiyar et al. (2019) in mice demonstrated that *SSPN* overexpression alleviated cardiomyopathy associated with Duchenne muscular dystrophy [62]. In the early 2000s, variants in another component of the DGC, δ-sarcoglycan *(SGCD),* were characterised in patients with familial and sporadic cases of DCM without any skeletal muscle disease [63].

The role of common variants causing DCM is supported by the generation of a GRS in over 300,000 participants with DCM. The GRS associated with CMR traits (reduced LVEF, increased LVEDV and LVESV). The penetrance of rare DCM-associated variants, the proportion of individuals with a variant that have DCM, is influenced by additional genetic and environmental influences. In this study, rare variant penetrance was modulated by common variants in the general population with a four-fold increased risk compared to the median [56]. In rare variant carriers, the GRS associated with a two-fold increase in DCM compared to the median. PheWAS of the GRS identified associations with cardiovascular phenotypes and obesity and portrayed an inverse relationship with HCM.

## Rare Variants Associated with Hypertrophic Cardiomyopathy

In general, rare variants (with a minor allele frequency < 0.1%) have larger effects on disease than their common variant counterparts [64, 65] (Graphical Abstract). Pathogenic, rare variants in twelve sarcomere-encoding genes are reported with definitive evidence for HCM (*MYBPC3, MYH7, MYL2, MYL3, TNNI3, TNNT2, TPM1, ACTC1*) in addition to four that have been newly upgraded by the ClinGen consortium (*CSRP3, TNNC1, ALPK3, PRKAG2*) but are not yet standard (Table 2) [18, 20, 35, 66]. The gene-disease associations were evaluated from published clinical genetic and experimental evidence. Genetic evidence included case-level data and variant analysis, while experimental evidence encompasses categories such as expression data and functional alterations. A gene was classified as "definitive" if it received a strong evidence score with consistent publications over at least three years and no contradictory evidence [8, 35].

**Table 2:** Eight genes classified with definitive evidence of causing HCM

Gene	Protein	Mode of inheritance	Literature supporting definitive classification
*MYL3*	Myosin light chain 3	Autosomal dominant	Ingles et al. [35] Clinical Genetics 2023 Josephs et al. [67]
*TNNI3*	Troponin I	Autosomal dominant	Ingles et al. [35] Clinical Genetics 2023 Josephs et al. [67]
*TNNT2*	Troponin T2	Autosomal dominant	Ingles et al. [35] Clinical Genetics 2023 Josephs et al. [67]
*TPM1*	Tropomyosin 1	Autosomal dominant	Ingles et al. [35] Clinical Genetics 2023 Josephs et al. [67]
*MYBPC3*	Myosin-binding protein C	Autosomal dominant	Ingles et al. [35] Clinical Genetics 2023 Josephs et al. [67]
*MYL2*	Myosin light chain 2	Autosomal dominant	Ingles et al. [35] Clinical Genetics 2023 Josephs et al. [67]
*ACTC1*	Alpha actin	Autosomal dominant	Ingles et al. [35] Clinical Genetics 2023 Josephs et al. [67]
*MYH7*	Myosin heavy chain 7	Autosomal dominant	Ingles et al. [35] Clinical Genetics 2023 Josephs et al. [67]

The genes with definitive evidence for causing HCM. Clinical Genetics 2023, ClinGen Consortium (https://www.clinicalgenome.org/)

Identifying rare variants enables precise patient risk stratification in family members. Sarcomeric variants are associated with an increased risk of mortality or experiencing major adverse cardiac events, primarily attributed to outcomes such as HF or arrhythmias. Individuals diagnosed with HCM who carry rare variants exhibit a notably heightened risk compared to those with HCM who do not have these variants [67].

Pathogenic variants in *MYBPC3* and *MYH7* account for most genotype-positive HCM cases [31], whereas the other genes account for < 10% of cases [68, 69]. *MYBPC3* encodes cardiac myosin-binding protein C (cMyBP-C), a key sarcomere protein that binds both actin and myosin [70]. *MYH7* is a sarcomeric gene encoding myosin heavy chain beta (MHC-β) and plays a crucial role in the contraction of muscle fibres [71, 72]. It is responsible for the ATP-dependent sliding movement of actin and myosin filaments which results in contraction [73].

The consequences of variants on the protein structure are important: protein-altering variants (denoted here as truncating [loss of function] plus non-truncating [e.g., missense variants]) in *MYBPC3* and non-truncating variants in the other seven genes are associated with HCM [74]. The most common variants in *MYBPC3* are frameshift, nonsense (stop gained), and essential splice-site variants, which result in premature termination codons [75]. Non-truncating variants (e.g., missense, inframe indels, start lost, and stop lost variants) account for roughly 15% of *MYBPC3* HCM, with clinical outcomes being similar in patients with truncating or non-truncating variants [76].

*MYL2* and *MYL3* encode myosin regulatory and essential light chains, respectively. [76] Compared to *MYH7* and *MYBPC3,* pathogenic variants in *MYL2* and *MYL3* as a cause of HCM are relatively rare [77, 78]. They present with variability in clinical manifestation and disease severity [79, 80]. *TNNT2* and *TNNI3* encode proteins belonging to the troponin complex. *TNNT2* encodes for troponin T within the cardiac muscle, which helps anchor the troponin complex on the actin-tropomyosin thin filaments and has a central role in the regulation of muscle contraction and relaxation [81]. *TNNI3* encodes the cardiac isoform of troponin I, the inhibitory subunit of the troponin complex, which prevents muscle contraction by inhibiting the actomyosin activity of the myosin-heavy chain [82, 83]. *TPM1* (tropomyosin 1) regulates the contraction of the sarcomere by controlling interactions between actin and myosin [84]. *ACTC1* encodes cardiac alpha-actin, which is a major component of the thin filaments and is responsible for the alignment and stability of thin filaments within the sarcomere [85].

Variants in other genes can cause rare syndromic disorders with isolated hypertrophy. Examples include variants in *PRKAG2, GLA, ALPK3, CACNA1C, DES, FHL1, FLNC, GLA, LAMP2, PRKAG2, PTPN11, RAF1, RIT1,* and *TTR*. Pathogenic variants in *PRKAG2*, encoding the gamma-2 regulatory subunit of adenosine monophosphate (AMP)-activated protein kinase (AMPK) [86], cause severe ventricular hypertrophy, electrocardiographic pre-excitation and conduction system disease [87]. These variants cause glycogen accumulation within cardiomyocytes, a disease of cardiac glycogen metabolism [87]. Lopez-Sainz et al. (2020) demonstrated an increased risk of adverse cardiovascular events such as atrial fibrillation, advanced HF, life-threatening arrhythmias, and sudden cardiac death in carriers with incomplete penetrance [88, 89]. A worsening prognosis was observed in those with abhorrent glycogen metabolism compared to disease caused by a sarcomeric variant [88]. Fabry disease is caused by recessive variants in the *GLA* gene resulting in reduced or absent activity of the enzyme alpha-galactosidase [90]. The altered enzyme activity results in glycophospholipid metabolism leading to the deposition of globotriasylceramide within the myocardium causing HCM as the main cardiac manifestation [90, 91]. However, unlike *PRKAG2* variants, Fabry disease is treatable with enzyme replacement therapy [88], and hence early recognition of its diagnosis is imperative to improve prognosis.

Rare variants associated with HCM are increasingly recognised with other phenotypes, such as restrictive cardiomyopathy. Restrictive cardiomyopathy presents with restrictive left ventricular filling due to impaired relaxation and compliance [92]. The genes with variants associated with restrictive cardiomyopathy include *TNNT2, ACTC1, MYBPC3, MYL2* and *MYL3*; all of which are associated with HCM [93]. Although uncommon, HCM can present similarly to primary restrictive cardiomyopathy, whereby patients have mild hypertrophy but severe diastolic dysfunction; coined HCM with ‘restrictive phenotype’. Notably, a missense variant in *MYL2* was found to increase the incidence of the restrictive phenotype by over one-third [94].

Understanding the estimated penetrance of variants is of utmost importance to inform risk stratification. Penetrance is the probability of disease given a risk variant. In patients, the penetrance of pathogenic variants in HCM was estimated in a recent meta-analysis from Topriceanu et al. (2024) as ~ 55% for *MYBPC3,* 65% for *MYH7*, 65% for *MYL2,* 32% for *MYL3*, and 60% for *TNNT2* and *TNNI3* [95]*.* With large datasets now available, the population penetrance of specific variants in cardiomyopathy-associated genes is now being estimated and is lower than that observed in the clinic [96].

## Rare Variants Associated with Dilated Cardiomyopathy

Rare variants have been identified that are associated with DCM (Table 3). [36, 97, 98] Jordan et al. (2021) used published results from genetic testing and experimental evidence to classify variants [36]. 12 genes including *LMNA, BAG3, TTN, MYH7* and *TNNT2,* have definitive evidence for causing DCM.

**Table 3:** Twelve genes classified with definitive evidence of causing DCM

Gene	Protein	Mode of inheritance	Literature supporting definitive classification
*BAG3*	BCL2-associated athanogene 3	Autosomal dominant	Jordan et al. 2021 [36] Seidel et al. 2021 [98] Mazzarotto et al. [97] Clinical Genetics 2023 Josephs et al. [67]
*DES*	Desmin	Autosomal dominant	Jordan et al. 2021 [36] Clinical Genetics 2023 Josephs et al. [67]
*DSP*	Desmoplakin	Autosomal dominant	Seidel et al. 2021 [98] Mazzarotto et al. [97] Josephs et al. [67]
*FLNC*	Filamin C	Autosomal dominant	Jordan et al. 2021 [36] Clinical Genetics 2023 Josephs et al. [67]
*LMNA*	Lamin A/C	Autosomal dominant	Jordan et al. 2021 [36] Seidel et al. 2021 [98] Mazzarotto et al. [97] Clinical Genetics 2023 Josephs et al. [67]
*MYH7*	Myosin heavy chain 7	Autosomal dominant	Jordan et al. 2021 [36] Seidel et al. 2021 [98] Mazzarotto et al. [97] Clinical Genetics 2023 Josephs et al. [67]
*PLN*	Phospholamban	Autosomal dominant	Jordan et al. 2021 [36] Mazzarotto et al. [97] Josephs et al. [67]
*RBM20*	RNA-binding motif protein 20	Autosomal dominant	Jordan et al. 2021 [36] Clinical Genetics 2023 Josephs et al. [67]
*SCN5A*	Sodium voltage-gated channel, alpha subunit 5	Autosomal dominant	Jordan et al. 2021 [36] Clinical Genetics 2023 Josephs et al. [67]
*TNNC1*	Troponin C	Autosomal dominant	Jordan et al. 2021 [36] Mazzarotto et al. [97] Clinical Genetics 2023 Josephs et al. [67]
*TNNT2*	Troponin T2	Autosomal dominant	Jordan et al. 2021 [36] Seidel et al. 2021 [98] Mazzarotto et al. [97] Clinical Genetics 2023 Josephs et al. [67]
*TTN*	Titin	Autosomal dominant	Jordan et al. 2021 [36] Seidel et al. 2021 [98] Mazzarotto et al. [97] Clinical Genetics 2023 Josephs et al. [67]

The genes have definitive evidence for causing DCM. Clinical Genetics 2023, ClinGen Consortium (https://www.clinicalgenome.org/)

*LMNA* encoding lamin, a component of the nuclear envelope, is one of the most common genes associated with DCM [99, 100]. This gene also has high population penetrance [99]. Both heterozygous and homozygous variants have been reported to result in DCM, with homozygous variants resulting in a fatal phenotype and a high rate of sudden cardiac death due to arrhythmias [100]. Typically, *LMNA*-related DCM has early onset arrhythmias followed by conduction abnormalities which can result in sudden cardiac death [101].

*BAG3*, encoding B-cell lymphoma-associated athanogene 3 or BAG cochaperone 3, has many crucial roles within cardiomyocytes; it maintains sarcomere integrity, regulates macroautophagy, promotes the expression of *BLC2* to maintain antiapoptotic properties, boosts mitochondrial quality, and regulates beta-adrenergic/L-type calcium channels [102]. Truncating variants in *BAG3* have been observed in DCM patients [97]. The loss of function likely causes destabilization of the Z-disc, impaired protein homeostasis leading to proteotoxicity, and increased susceptibility to apoptosis [66]. Pathogenic variants in *BAG3* present with variability in clinical manifestation and age of onset [102]. Male sex, decreased left ventricular ejection fraction, and large ventricular end-diastolic diameter, are associated with adverse outcomes in those with pathogenic variants in *BAG3 *[103].

A particular common missense variant within *BAG3*, characterized by the substitution of cysteine with arginine at position 151 of the BAG3 protein (C151R; rs2234962) [104], demonstrates a potential cardioprotective function in GWAS of DCM, HF, and ejection fraction [108] with risk for HCM [52]. The variant is associated with proteins involved in the maintenance of myofibrillar integrity and causes improved response to proteotoxic stress [105]. Comprehending the functional alteration in BAG3 associated with this cardioprotective effect holds promise for devising therapeutic interventions aimed at targeting the C151R variant specifically. Such insights into genetic modifiers could pave the way for the development of tailored treatments, including the overexpression of this *BAG3*:p.C151R variant to leverage its potential benefits.

*TNNT2* is associated with an early, relatively aggressive form of DCM [106]. Likewise, *MYH7* is associated with an early age of onset, and HF is observed more commonly than ventricular arrhythmias in these patients [100]. Truncating variants in *FLNC* have been recently associated with DCM [107]. *FLNC* codes for the cytoskeleton protein filamin C, which functions as an actin crosslinking protein and anchors sarcolemma proteins to the cytoplasm to maintain the structural integrity of the sarcomere [108]. Studies of *FLNC* in DCM have been limited by a small number of participants due to the use of targeted sequencing methods that excluded analysis of the gene [109, 110]. Ader et al. (2019) found that among 300 patients with known DCM, 10 carried a mutation in *FLNC* (3%) [119]. Patients carrying a pathogenic variant in *FLNC* have been reported to have a high risk of sudden cardiac death and a more malignant clinical course [109, 110]. Pathogenic variants in *FLNC* and *LMNA* are associated with a significant increase in myocardial fibrosis [100, 107] and result in a worse prognosis regarding mortality and life-threatening arrhythmia compared to other genetic variants [51, 110–114]. The 2023 European Society of Cardiology guidelines also highlight variants in *DSP* (encoding desmoplakin) [115], *RBM20* (encoding RNA-binding motif protein 20) [116], *PLN* (encoding phospholamban) [117] and *TMEM43* (transmembrane protein 43)*,* with a substantially higher risk of arrhythmias. Variants in *TMEM43* are more strongly associated with arrhythmogenic cardiomyopathy (ARVC or ACM) [118–121].

## Overlapping and Opposing Genetic Factors of HCM and DCM

Genes with rare variants that have definitive or moderate evidence curated by experts for both HCM and DCM have been identified (e.g., *TNNC1, TNNI3, TNNT2, MYH7, TPM1, ACTC1, JPH2, PLN*). *PLN* is associated with “intrinsic cardiomyopathy” as while most variants reported are associated with DCM, HCM has also been reported [67]. Missense variants in *MYH7* are a common pathogenic cause of DCM and HCM, with the current understanding that an individual missense variant in *MYH7* can convey risk for DCM or HCM, but not both [122].

Pathogenic *MYH7* variants cause cardiomyopathies primarily due to decreased (DCM) or increased (HCM) sarcomere force generation [66]. Variants in *MYH7* causing HCM produce an abnormally activated protein that incorporates into the sarcomere as a ‘poison peptide’. Such dominant-negative variants associated with HCM are concentrated in the myosin head domain (amino acid residues 181–937 [66]; including the motor domain) of the *MYH7* gene, whereas those associated with DCM may occur across the gene. DCM-associated *MYH7* missense variants likely reduce the passive stiffness of myofibrils and are deficient in force generation and force-holding capacity [66, 68]. Variants in the globular head can affect the formation of binding sites for actin and cross-bridges for contraction [123]. Variants can either directly affect motor function or can impact on myosin "interacting head motif" and impair kinetics [66]. A study by Ujfalusi et al. (2018) focused on pathogenic variants within the myosin head domain of *MYH7,* selecting five DCM-linked and two HCM-linked variants [124]. The authors concluded that pathogenic variants of *MYH7* caused discrepancies by altering individual steps in the ATPase cycle. Compared to wild-type or HCM variants, myosin with DCM-associated variants had a lower duty ratio; the motor domain remained bound to actin for a decreased proportion of the ATPase cycle. As a result, less force was generated and there was reduced force-holding capacity in DCM mutants, while HCM mutants had greater force-holding capacity. However, the results relied on the mechanisms of a fully activated cross-bridge, while cardiac sarcomeres operate at submaximal activation.

Recent HCM and DCM genome-wide association studies (GWAS) of common DNA variants (with individually small effects on disease) have identified overlapping loci for cardiomyopathies. Tadros, Francis, Xu, Vermeer, et al. (2021) [155] showed an opposing relationship between HCM- and DCM-associated common DNA variants and left ventricular measures of structure and function through analyses of the common variants [155]. Genetic correlation between the left ventricular measures of structure and function had divergent relationships with HCM and DCM (most strikingly with end-systolic volume [LVESV; DCM+, HCM-], ejection fraction [LVEF; HCM+, DCM-], and measures of strain [HCM+, DCM-]). They identified variants associated with both cardiomyopathies that showed opposite directions of effect in HCM versus DCM [23]. The authors concluded that this indicates that genetic loci underlying the variability of left ventricular function in the general population may be differentially involved in susceptibility to HCM and DCM. An assessment of cumulative variation in cardiomyopathy-associated genes also identified oppositions between HCM and DCM [125] and a pheWAS of HCM-derived GRS identified a protective relationship for HF [126].

Further to the described opposing relationships between HCM and DCM with variants in *BAG3* and *MYH7*, an example of an opposing common variant is a SNP (rs3176326) in *CDKN1A* [23]*.* The protein is involved in cell death and cell cycle arrest, and knockout mice can regenerate damaged or missing tissue. The minor A allele of the variant is associated with decreased muscle expression (GTEx browser); decreased cell death and thus more cell growth. This increase in cell cycling increases HCM risk and decreases DCM and HF risk. This opposing relationship provides potential support for a protection theory, where variants influencing non-obstructive HCM may provide some protective effect against DCM and HF risk at the cellular level.

## *FHOD3*: A Reoccurring Hypertrophic Cardiomyopathy-Associated Gene

The FHOD3 (formin homology 2 domain containing 3) protein regulates sarcomere organisation and myofibrillogenesis and maintains the contractile apparatus in cardiomyocytes [128, 129]. The burden of protein-altering variants in *FHOD3* is significantly increased in patients with HCM compared to controls [130]. In 2020, Ocha et al. discovered copy number variants (CNVs; copies of a segment of DNA that vary in number) in *FHOD3* in patients with HCM that were not identified in controls [131]. A GWAS by Wooten et al. (2013) identified an *FHOD3* intronic variant found to more than double the risk of HCM (rs516514, *P* = 1.25 × 10^–7^) [49, 132] that was in linkage disequilibrium with the sentinel missense SNP FHOD3-V1151l (rs2303510; *P* = 1.76 × 10^–9^). *FHOD3*-V1151l was also associated with HCM two further cohort analyses [132]. A genetic analysis of *FHOD3* variants in a Chinese population identified *FHOD3* candidate variants as independent predictors for cardiovascular death and all-cause death in HCM (adjusted HR, 3.02; 95% CI, 1.09–6.85; P = 0.035) [131]. A more common, intermediate effect variant in *FHOD3* (p.Arg637Gln; gnomAD NFE AF 0.03%) has been associated with a clinically aggressive course and an earlier presentation in homozygous carriers, consolidating the association between *FHOD3* and the development of HCM [133].

## *TTN*: Recent Evidence for a Major Cause of DCM

Titin is the largest human protein spanning from the M-line to the Z-disc of the cardiac sarcomere (Fig. 1). Truncating variants in the *TTN* gene (TTNtvs) have been implicated in numerous cardiomyopathies, including DCM and other environment-influenced cardiomyopathies such as peripartum, alcohol-induced, and chemotherapy-induced, cardiomyopathy [134–138]. Heterozygous TTNtvs are the most common genetic cause of DCM (15%-20% of all patients with DCM) and are found in about 2% of the general population [137, 139, 140]. Due to the size of the gene, analyses of TTNtvs are complicated by the rarity of individual- and family-specific variants so effects are usually assessed in aggregate.

Roberts et al. (2015) used data from over 5,000 patients to establish that TTNtvs in constitutive exons expressed in the heart were the most pathogenic [138]. Importantly, if the TTNtvs can be ‘bypassed’ by alternate splicing, its effect on the cardiac phenotype is limited [141]. Thus, TTNtvs in the A-band have a higher odds ratio for DCM than those in parts of the highly spliced I-band [137, 142]. Notably, the position of the TTNtvs did not correlate with the patient’s age at the time of transplant or the pretransplant left ventricular function in a study of DCM hearts removed at transplantation [143]. Studies identifying modifiers of TTNtvs are needed.

The mechanisms by which TTNtvs cause cardiomyopathy has been difficult to decipher largely due to the size of the gene. Suggested pathomechanisms included nonsense-mediated decay of TTNtvs mRNA and haploinsufficiency (the remaining functional copy of the gene is not adequate for normal function) [143–145]. The ‘poison-peptide’ theory, which states that truncated titin (tr-titin) hampers sarcomeric function, was considered a less likely explanation as tr-titin was not shown to be present in adult DCM hearts [146]. However, this was recently shown by McAfee et al. (2022) [143] who also observed lower amounts of wildtype titin in mutant hearts, indicating haploinsufficiency occurring in parallel.

Fomin et al. (2022) further revealed that the tr-titin proteins are sequestered in intracellular aggregates that dysregulate protein quality control pathways (PQCs), mainly the ubiquitin pathway [145]. There was increased ubiquitin degradation of wild-type titin in mutant hearts, which may cause the associated poor contractility. Inhibition of PQCs using proteasome inhibitors increased levels of both tr-titin and wild-type titin, resulting in increased contractility of human induced pluripotent stem cell-derived cardiomyocytes (hiPSC-CMs) derived from patients with TTNtvs.

Evidence from these studies suggests that TTNtvs cause DCM through both haploinsufficiency and the ‘poison peptide’ theory. The two functional studies analysed samples from patients with end-stage HF and future research with samples from patients at different stages of disease may provide greater insight into which mechanisms require treatment prioritisation and which TTNtvs have highest penetrance. As the penetrance of TTNtvs is incomplete, even in pathogenic locations, many individuals do not develop cardiomyopathy [138] and our understanding of this is required for TTNtvs to become more clinically actionable.

In aggregate, missense variants in *TTN* are not enriched in DCM patients even when predicted to be deleterious [147]. However, there is evidence of segregation (where variant carriers present with disease in affected families) for specific *TTN* missense variants in DCM (c.2926 T > C (p.Trp976Arg); c.533C > A (p.Ala178Asp); and c.11674 T > A (p.Cys3892Ser)) [66, 148]. The molecular mechanisms by which these variants may predispose individuals to DCM requires investigation.

## Discussion: Future Directions and Therapeutic Opportunities

Over 100 genetic loci have been identified with common variants associated with HF and tens of genes with protein-altering variants have definitive evidence for causing cardiomyopathy-induced HF. The different HF subgroups have heterogeneous genetic influences; HFpEF has the lowest estimated heritability and associates with few genetic loci compared to HFrEF and less specific HF groupings. Improved sample size and patient stratification will aid our understanding of this in future studies. With this improved understanding of the genetic factors influencing HF, cardiac and extracardiac (e.g., kidney, vascular, and metabolic) tissue components as well as cellular and risk factor-associated molecular mechanisms are being identified as treatment targets.

Some of the genes with definitive evidence of rare, protein-altering variants causing cardiomyopathies and HF are identifiable at GWAS (e.g., *BAG3, TTN, MYBPC3, FLNC, PLN*), however, the absence of others is likely due to statistical power. With more whole genome sequencing, a holistic approach to discovering novel, common variant loci with confirmatory assessments of rare variants at the same locus via a burden analysis may provide further confirmation of which loci would be most important for targeted efforts for future HF therapy and would reflect the complex and diverse genetic architecture of cardiomyopathies [149, 150].

The analyses of larger structural variants (such as CNVs in *FHOD3*) and combinations of variants (oligogenic inheritance) may account for a proportion of cardiomyopathy that is genotype negative [129]. One crucial challenge in treating patients with HCM and DCM is ascertaining an early diagnosis to impose primary prevention of HF. The use of GRS composed of SNPs associated with DCM and HCM at GWAS may have clinical potential as diagnostic biomarkers [151, 152] and may be leveraged clinically for patients who are carriers of known pathogenic variants to predict the severity and prognosis of cardiomyopathies [153]. Notably, controlled diastolic blood pressure reduces the risk of developing sarcomere-negative HCM, a condition influenced by common variants and challenging to diagnose at an early stage [27]. With our improved understanding of the common and rare genetic influences over HF, treatments targeting the mechanisms of action of the largest effect genetic loci are required.

With increasing knowledge of the genes implicated in cardiomyopathy and subsequent HF, the use of multiomic analyses to decipher the molecular mechanisms by which such genetic variants cause cardiac dysfunction may enable disease-modifying HF therapeutics to be developed. Furthermore, single-cell transcriptomic and proteomic analyses of HF heart samples may help identify potential biomarkers of prognostic significance, enabling appropriate risk stratification and personalised management of patients with HF [154, 155]. The DCM heart atlas is a point of reference here [156].

CRISPR-Cas 9-based gene editing holds the potential to reverse diseases caused by specific genetic variants. It may be instrumental in managing cardiomyopathy-induced HF caused by rare variants and pre-clinical models have shown benefit in reversing TTNtv-induced contractile dysfunction [145]. A recent study using adenine base editors and Cas-9 to inactivate the pathogenic myosin R403Q allele in mice prevented the development of HCM [157, 158]. However, results from more extensive trials with longer follow-up will be keenly awaited as well as pipelines for high throughput production for rare variants found in small numbers of patients.

Pharmacogenetic studies in HF have mainly assessed variants affecting the sympathetic nervous system response [159–161]. Improved understanding of how genetic variants influence response to HF therapy could greatly improve outcomes by better prediction of patients unlikely to respond to the therapy. Trial cohorts provide the ideal data source for these studies [162]. Pharmacogenetic studies looking at the effect of common variants in response to SGLT2 inhibitors and angiotensin receptor neprilysin inhibitors may provide clinically valuable information. Similarly, genotyping of *CYP2C19* in patients with HCM treated with mavacamten, a novel cardiac myosin inhibitor, would aid dose tailoring and may improve outcomes [163]. Whether HFpEF patients benefit from Mavacamten is currently being evaluated (NCT04766892) and may guide future therapy in HFpEF [164]. The benefit may be most in HFpEF patients with a shared genetic architecture with HCM.

Further studies are needed to assess the impact of the coinheritance of multiple variants on disease development, severity and their subsequent impact on prognosis and response to therapy. Whether there are recurrent combinations of genetic variants in an oligogenic fashion, remains elusive. Allelic imbalance, where allele expression is altered for one allele compared to the other, may also contribute to the incomplete penetrance and variable expressivity observed in cardiomyopathies [165, 166].

Estimates of the prevalence of cardiomyopathies and genetic studies to date are dominated by European participants. Biases aside, the linkage disequilibrium within participants of European ancestry allows for improved statistical power in identifying variants that are associated with disease at GWAS. Large biobanks including patients of more diverse ancestry are required to improve the generalization of genetic results across the globe and for GRS to be applied globally. Importantly, future multi-ancestry biobanks would allow for improved curation of rare variants identified in non-European ancestries as it is likely some variants that are observed rarely in European patients are common in other ancestries and are less pathogenic than previously expected [167].

The genetic association analyses of HF and cardiomyopathies contribute to our understanding of incomplete penetrance. To improve diagnostic yield, our understanding of penetrance for individual variants is required to fully utilize the promises of genetics for cardiovascular disease [168, 169]. Eventually coupling genomics with metabolomics, transcriptomics and proteomics, will facilitate a better understanding of the molecular mechanisms by which variants cause cardiomyopathy and subsequent HF. Incorporation of genetics into multiparametric risk stratification models for cardiomyopathy patients with future penetrance estimates will facilitate more personalised management, better selection of patients for advanced therapies, and improve patient outcomes.

## Conclusion

The advent of next-generation sequencing and GWAS has enhanced our understanding of the genes involved in HF. Common and rare genetic variants have been identified that are associated with HF as well as the risk factors and conditions that progress to HF. Recent GWAS and investigations into the biological mechanisms of TTNtvs have aided our understanding of the genetic underpinnings of HF and cardiomyopathy. Future work is required to validate the genes implicated in HF, identify novel therapeutic targets, and link specific variants to clinical outcomes through estimates of penetrance.
